# Vasodilator stress cardiovascular magnetic resonance imaging in post-orthotopic heart transplant recipients: evaluation of safety, hemodynamics, and myocardial perfusion

**DOI:** 10.1186/1532-429X-18-S1-P107

**Published:** 2016-01-27

**Authors:** Akhil Narang, Mita Patel, Victor Mor-Avi, Savitri E Fedson, Roberto Lang, Amit R Patel

**Affiliations:** grid.170205.10000000419367822Section of Cardiology, Department of Medicine, University of Chicago, Chicago, IL USA

## Background

Long-term survival after orthotopic heart transplant (OHT) is limited by coronary allograft vasculopathy and its associated myocardial perfusion abnormalities. Little is known how post-OHT patients respond to vasodilator stress cardiovascular magnetic resonance imaging (vsCMR). This study aimed (1) to evaluate the safety and hemodynamic response of post-OHT patients undergoing vsCMR with regadenoson and (2) to determine whether these patients have abnormalities in myocardial perfusion detectable by vsCMR.

## Methods

We studied 50 subjects, including 20 patients post-OHT and 30 controls (10 healthy volunteers and 20 patients with known myocardial infarction [MI]), who underwent vsCMR (1.5T scanner, Achieva, Phillips). Short-axis CMR images of the left ventricle (LV) were obtained during first pass of gadobenate-dimeglumine (0.075 mmol/kg at 4 ml/sec) for approximately 50 consecutive heartbeats. Images were acquired using a hybrid gradient echo/echo planar imaging sequence one minute after administration of regadenoson 0.4 mg and then repeated 15 minutes after reversal with aminophylline (125 mg). Hemodynamic data and side-effects and were recorded in all patients. Time-intensity curves generated from stress and rest perfusion images were used to determine myocardial perfusion reserve index (MPRi), which was calculated as the up-slope ratio of stress to rest (normalized to the LV cavity and rate-pressure-product). For the MI group, MPRi was assessed in a segment remote to the ischemic/infarct territory based on late gadolinium enhancement and invasive coronary angiography. Differences between patient groups were tested using the Mann-Whitney U test.

## Results

**Figure 1:**
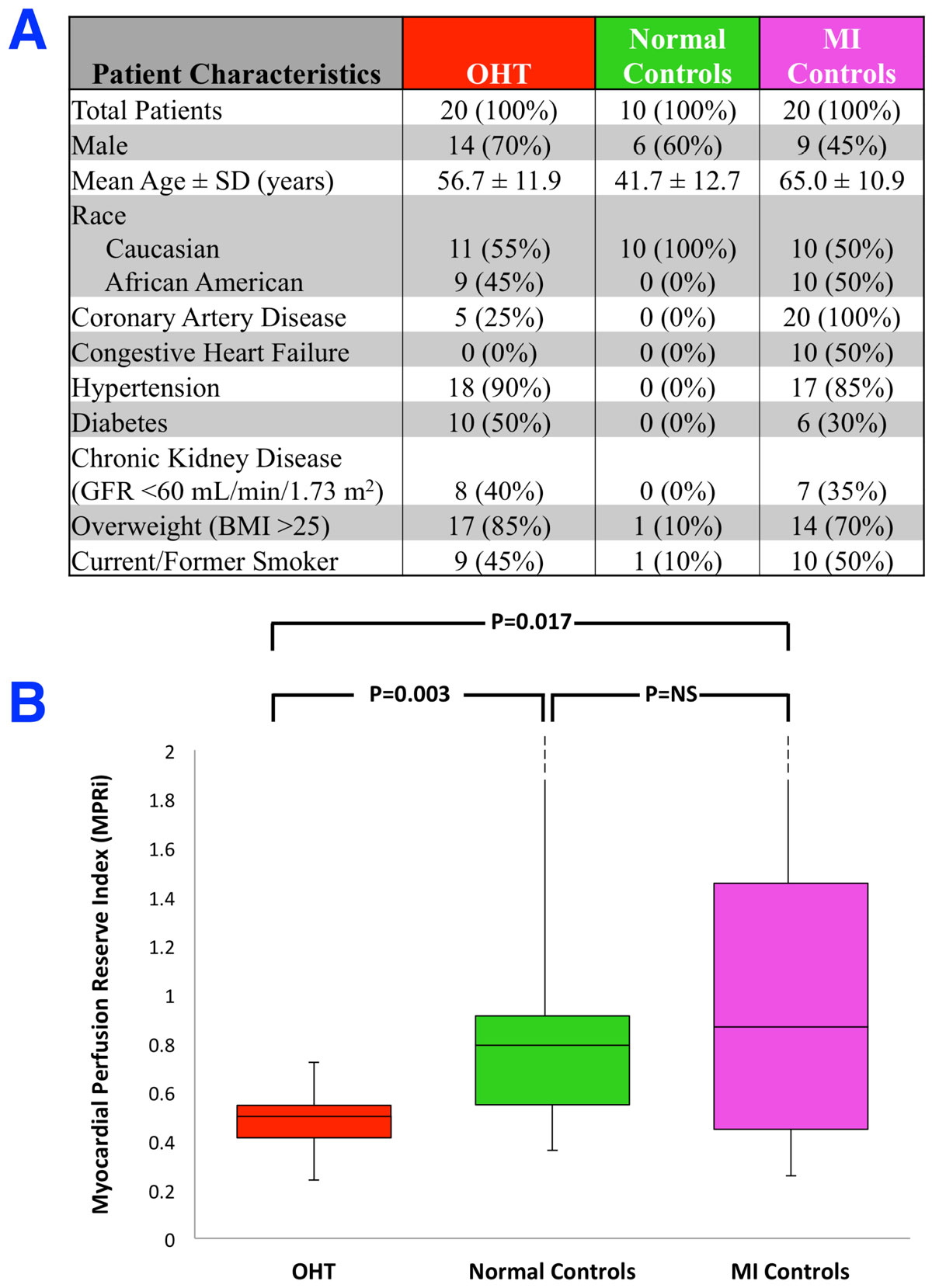
**A. Patient characteristics including risk factors for cardiovascular disease**. **B.** Box-and-whisker plot of myocardial perfusion reserve index (MPRi) for 20 OHT patients and 30 controls (10 normal volunteers and 20 patients with known myocardial infarction).

Patient characteristics are shown in Figure 1A. The time from OHT to vsCMR was 8.1 ± 4.1 years. The most common side-effects from regadenoson included dyspnea, flushing, headache, chest pain, and palpitations with no significant increase in overall side-effect profile in post-OHT patients when compared to controls. The hemodynamic (blood pressure and heart rate) response to regadenoson in post-OHT patients did not differ from control patients. None of the patients experienced arrhythmias. All patients were asymptomatic at the conclusion of the exam. The median MPRi was reduced in post-OHT patients (0.50) when compared to normal (0.79) and MI groups (0.87) (P = 0.003 and 0.017, respectively). There was no difference in the MPRi between normal and MI groups (Figure 1B).

## Conclusions

Post-OHT patients can safely undergo vsCMR imaging and experience a similar hemodynamic response to control patients. MPRi was significant reduced in post-OHT patients when compared to healthy volunteers and patients with known prior MI. Future studies examining the mechanisms of the reduced MPRi in post-OHT patients are warranted.

